# Clinical Holistic Medicine: Holistic Treatment of Mental Disorders

**DOI:** 10.1100/tsw.2005.50

**Published:** 2005-05-23

**Authors:** Søren Ventegodt, Niels Jørgen Andersen, Shimshon Neikrug, Isack Kandel, Joav Merrick

**Affiliations:** ^1^ Nordic School of Holistic Medicine and Quality of Life Research Center, Teglgårdstræde 4-8, DK-1452 Copenhagen K, Denmark; ^2^ The Scandinavian Foundation for Holistic Medicine, Sandvika, Norway; ^3^ Norwegian School of Management, Sandvika, Norway; ^4^ Faculty of Social Science, Academic College of Judea and Samaria, Ariel, Israel; ^5^ National Institute of Child Health and Human Development and Center for Multidisciplinary Research in Aging, Faculty of Health Sciences, Ben Gurion University of the Negev, Beer-Sheva, Office of the Medical Director, Division for Mental Retardation, Ministry of Social Affairs, Jerusalem, Israel

**Keywords:** quality of life, QOL, philosophy, human development, holistic medicine, public health, holistic health, holistic process theory, life mission theory, group therapy, mental disorders, schizophrenia, responsibility-for-life scale, Denmark

## Abstract

We believe that holistic medicine can be used for patient's with mental health disorders. With holistic psychiatry, it is possible to help the mentally ill patient to heal existentially. As in holistic medicine, the methods are love or intense care, winning the trust of the patient, getting permission to give support and holding, and daring to be fully at the patient's service. Our clinical experiences have led us to believe that mental health patient's can heal if only you can make him or her feel the existential pain at its full depth, understand what the message of the suffering is, and let go of all the negative attitudes and beliefs connected with the disease. Many mentally ill young people would benefit from a few hours of existential holistic processing in order to confront the core existential pains. To help the mentally ill patient, you must understand the level of responsibility and help process the old traumas that made the patient escape responsibility for his or her own life and destiny. To guide the work, we have developed a responsibility scale going from (1) free perception over (2) emotional pain to (3) psychic death (denial of life purpose) further down to (4) escape and (5) denial to (6) destruction of own perception and (7) hallucination further down to (8) coma, suicide, and unconsciousness. This scale seems to be a valuable tool to understand the state of consciousness and the nature of the process of healing that the patient must go through.

## INTRODUCTION

In this paper, we will demonstrate how to heal some mental disorders through love (or intensive care), trust, holding, and processing[1,2,3,4,5,6,7,8,9] using the general holistic theory of mental diseases. Many physicians and psychiatrists from the biomedical paradigm seem to believe that mental diseases cannot be healed, but the biomedical approach can alleviate the symptoms and give many patients the possibility to lead a normal or almost-normal life. The recovery literature on the spontaneous healing of mental disease, with the recovery of schizophrenia as the most radical example, show that about one in four of even the most severe mental patients will eventually be well again without the intervention of a physician. The recovery literature show several kinds of recovery from schizophrenia[10], the most interesting being full recovery happening in one study in 13.7% of the patients after 5 years[11] and in about 25% long term[12,13] in the western countries and quite surprisingly much more in the third world[14]. Coming from the holistic paradigm, we believe strongly that everyone has huge hidden resources and the organism contains strong self-healing powers, which can be mobilized by the intervention in the holistic medical clinic. Before we give a series of examples of this induction of spontaneous recovery and holistic healing, we would like to introduce the reader to our previous work in this field. The paper will explain, through a number of case stories, why holistic healing can work for the patient with mental illness.

## WHAT IS INSANITY?

In our way of looking at holistic medicine, we define insanity as ***the degeneration of our state of consciousness and the subsequent degeneration of our behavior***. From a theoretical perspective, it is caused by the repression of our purpose of life[5]. Spiritual, mental, physical, and sexual character[15] are also repressed, and so are the mental, emotional, and physical strengths[7] together with gender and sexuality[16]. We aim to integrate all these dimensions in the much simpler perspective that insanity is caused by ***the loss of responsibility for our own existence***; recovery from mental illness is, therefore, a question of reassuming responsibility for life.

In theory, there are three dimensions of existence to be responsible for: love, power, and joy[7]. Love is about purpose of life or our personal mission. Power is the personal power of mind, feelings, and body. Joy is the dimension of gender, character, and sexuality. If the three dimensions collapsed randomly, or if they collapse in parallel, we would somewhat have the pattern of a spiral or whorl, going from full responsibility for the three key areas to zero (see Fig. 1). From our clinical findings, this is not the pattern we normally observe; it seems that first love is lost, then the feelings and the rational mind, and finally, sexuality and life itself. Another interesting finding is that the dimension is normally not lost in one trauma, but the person's own freedom or permission to use the dimensions are withdrawn before the dimensions are completely lost in a qualitative shift of the patient's existence, changing both state of consciousness, perception, and behavior (see Fig. 2). It therefore seems possible to create a one-dimensional scale of existential responsibility (see Table 1).

The different qualities of perceptions related to mental diseases can be understood as the gradual loss or recovery of responsibility and permission, gradually de/reconnecting the patient with reality and urge. As a general rule, first love is lost, then mind and feelings, and at last, the fundamental coherence in life itself. Permission can be defined to the level of giving in to the experienced urges (the urge of love, the urge of mind and feelings, the urge of sex and biological life).

This scale can be further elaborated into a highly structured and logical scale of responsibility (Table 2) that can be further elaborated into a detailed scale of the parallel decay of existential responsibility, perception, and behavior (Table 3). This scale has proven clinically useful when we, as holistic physicians, want to help patients to assume responsibility; as the scale illustrates the normal pathogenesis of mental diseases. We have tested the hypothesis that we can induce healing — salutogenesis with the famous expression of Aaron Antonovsky — using the scale as a staircase upwards to full health for our patients. The responsibility scale is, thus, guiding the holistic physician's induction of the mentally ill patient's recovery[2].

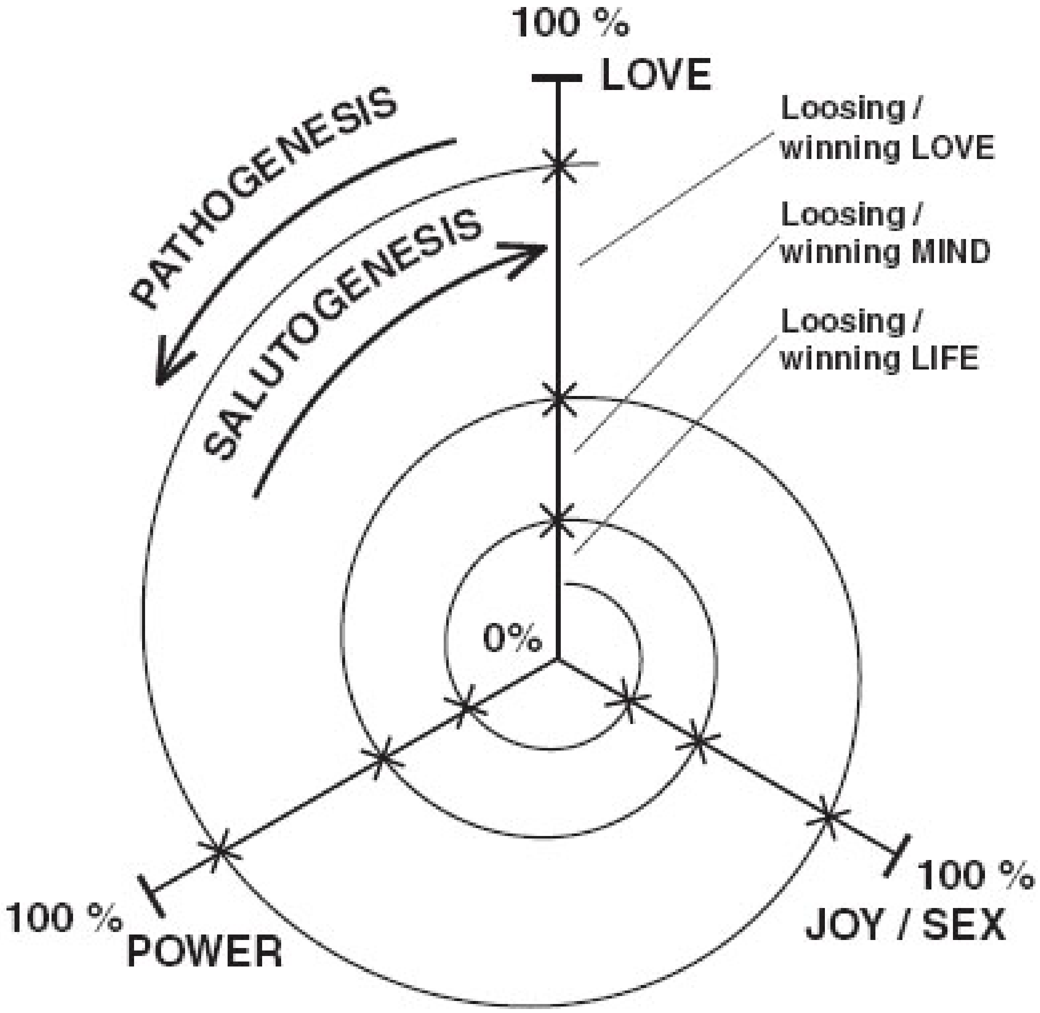

**FIGURE 1.** How the loss of existential responsibility causes insanity — theoretical model. If the three dimensions of love, power, and joy (from theory of talent[7]) are collapsed randomly, or if they collapse in parallel, we would somewhat have the pattern of a spiral or whorl, going from full responsibility for the three key dimensions down to zero. Responsibility can be defined as responsiveness to all experienced gaps, the gaps being between self and others, in self or in others, or even on the group level (in "the space"). ("X" represents a traumatic life event.)

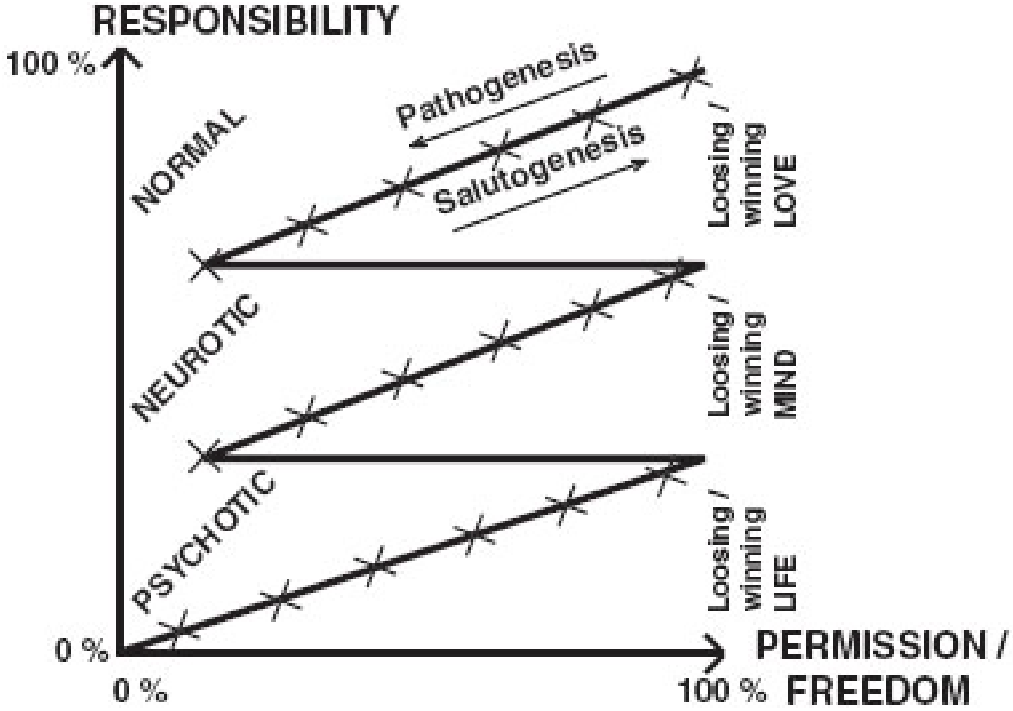

**FIGURE 2.** How the loss of existential responsibility causes insanity — clinical findings. Clinical findings, where patients are regressing back to their childhood traumas in holistic existential therapy, demonstrate that love is lost first, then power, and finally, “sexuality” in its most primitive sense. Every dimension of love, power, and joy/sex is lost gradually, until it goes under in a qualitative shift, changing state of consciousness, perception, and behavior at the same time. (“X” represents a traumatic life event.)

**TABLE 1**
**Pathogenesis of Mental Diseases Seen as an Eight-Step Responsibility-for-Life Scale**

| | |
|---|---|
| 1. | Free perception (fully conscious and happy) |
| 2. | Painful perception (perceiving something unwanted) |
| 3. | Psychic death (overwhelmed by emotional pain) |
| 4. | Escape (changing perspective) |
| 5. | Denial (lying) |
| 6. | Destruction of own perception ("blacking out") |
| 7. | Hallucination (seeing what is wanted instead of reality) |
| 8. | Suicide, unconsciousness, coma, physical death |

When a person meets resistance and bad luck, life turns painful; when it turns to painful, we escape; when we cannot escape, we lie; when we cannot lie, we black out; when we cannot black out because the roof is falling down on our head, we hallucinate; and when this is impossible, we die (i.e., commit suicide). The theory of pathogenesis is also, when read bottom up, the path of salutogenesis and, thus, helpful when we work with low-responsibility patients, which are normally almost impossible to cure and help, i.e., suicidal patients. Some cancer patients and other somatically ill patients who are actually dying seem to be awfully low on this scale too, and might be helped in the same way, as there is a psychosomatic element in most disease.

**TABLE 2**
**Responsibility × Permission Diagram**

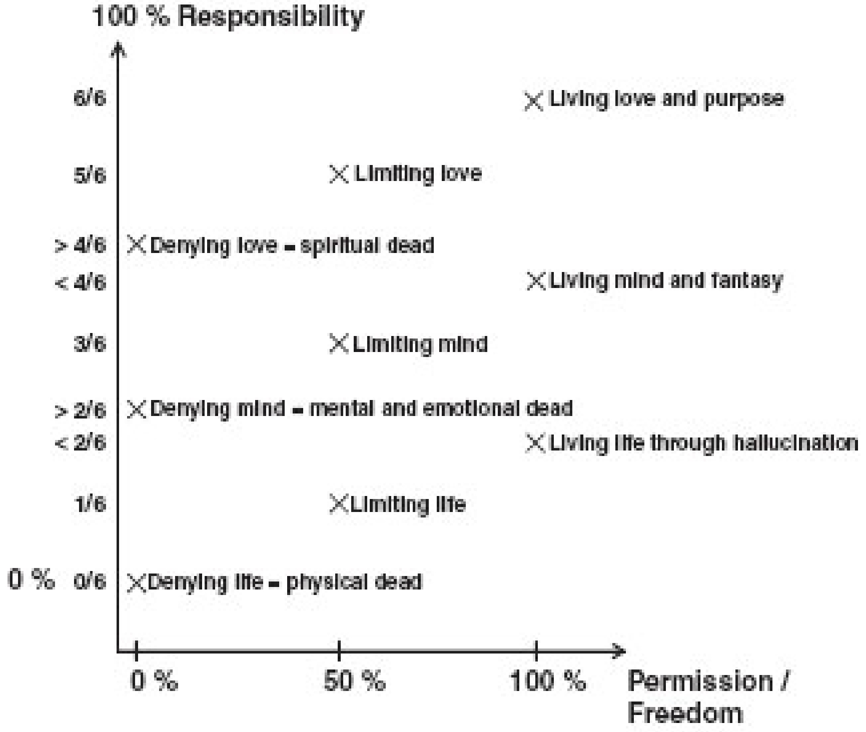

The different qualities of perceptions related to mental diseases can be understood as the gradual loss or recovery of responsibility and permission, gradually de/reconnecting the patient with reality and urge. First love is lost, then mind, and at last, the fundamental coherence with life itself.

As we shall illustrate with clinical examples below, this seems to work surprisingly well, supporting the hypothesis that the fundamental causal elements of mental disease, which of course coexist with genetic vulnerability, are the traumatic life events that make the human want to escape existential responsibility when overwhelmed by painful feelings and emotions. The patient then first enters a state of no-love, then it goes to ego-death; it reappears in the head as a reasonable although neurotic being. When overwhelmed again, it starts to produce lies and ends completely blacked out and psychotic, before going further into a blossoming hallucinatory state and further down to "negative symptoms" (not even hallucinating any more) — helplessness, shock, unconsciousness, suicide, coma, and death.

Theoretically, the scale is easy to understand, as it is a product of the gradual loss of first love, then mind, and finally, life itself. As the patient goes "down under", he or she loses the ability to love, feel, think, be, enjoy, do, and live as a sexual being. At a first glance into the mentally ill patient's personality, mind, and subconscious, the deterioration of existence including quality of life, health, ability, character, gender, meaning, and coherence with the world happens rather chaotically as the negative and self-destructive decisions are taken. Interestingly, there seems to be yet another pattern, the famous pattern of multiple personalities, a pattern obvious in some types of schizophrenia and much less obvious in most other patients until looked for. This pattern is illustrated in Fig. 3. As the different parts return to normal during therapy, they integrate and merge into the wholeness of the person.

**TABLE 3**
**Responsibility-for-Life Scale**

| Degree of Responsibility for Your Own Existence (Estimated Percentage) | State of Consciousness (Many Substates Exist) | Behavior (Other Patterns Might Exist) |
|---|---|---|
| 100% responsibility<br>**Mentally healthy** | Present, fully aware, interpreting the world according to your purpose of life | Succeeding, playing |
| 90–80% | Emotional pain (denying and repressing the feelings) | Fighting, attacking |
| 66%<br>**Neurotic** | Emotionally overwhelmed, psychic death (denying the purpose of life) | Fighting, defending |
| 50% | Escaping from here and now | Flight, running |
| 40% | Cannot escape, denying here and now | Freezing, helplessness |
| 33%<br>**Psychotic** | Destruction of the perception (wiping out, “blackness”, “closing eyes”, denying the mind) | Shocked, numb, lame |
| 20–10%<br>**Hallucinating**<br>(substituting perception) | Dreaming (perception and behavior not related to the outer world) | Dream state |
| 0% responsibility<br>**Dead** | Unconscious, in coma (denying the body) | Physically dying, suicidal, evil and destructive |

The scale describes how existential responsibility — seen from inside (the state of consciousness) and outside (the behavior) — is first lost and then found as the patient climbs the ladder of hallucination, blacking out, denial, escape, psychic death, unbearable emotional pain, to freedom of perception. To rehabilitate a psychotic patient in a hallucinatory state of consciousness, you need to help him/her confront the choking trauma that originally motivated the escape into hallucination; in doing this, you must carefully avoid pushing them deeper down into suicide (see text).

The loss of sanity during a traumatizing personal history and the recovery during holistic existential therapy of love, mind, and life itself is illustrated excellently with the case of the patient Anna[17], who took literally hundreds of negative life-denying decisions as she was abused during her childhood, and recovered from a borderline state as she confronted her different inner parts and the repressed emotions and decisions connected to them and let go of these decisions. To help the most severely ill patients, the advanced toolbox of holistic medicine must be taken into use[18]. Be aware that this takes years of training in clinical holistic medicine to be able to use the most powerful tools. What is important is to take care of the patient’s safety, as one repressed inner part might want to commit suicide and this part must be confronted with extreme attention; we call this “to push the patient down”. It is a necessary part of deep existential therapy, as the repressed parts cannot be healed in the patient without this confrontation, but the existential crises reappearing to the surface of the patient’s consciousness must be fully addressed.

The transformations of perception in this scale can be understood if we look at a two-dimensional presentation, with permission and responsibility as the two axes (Fig. 1). We see the theoretical background for the responsibility scale, namely the repression of the three existential dimensions of man (cf. the theory for talent[7]: love, power, and joy).

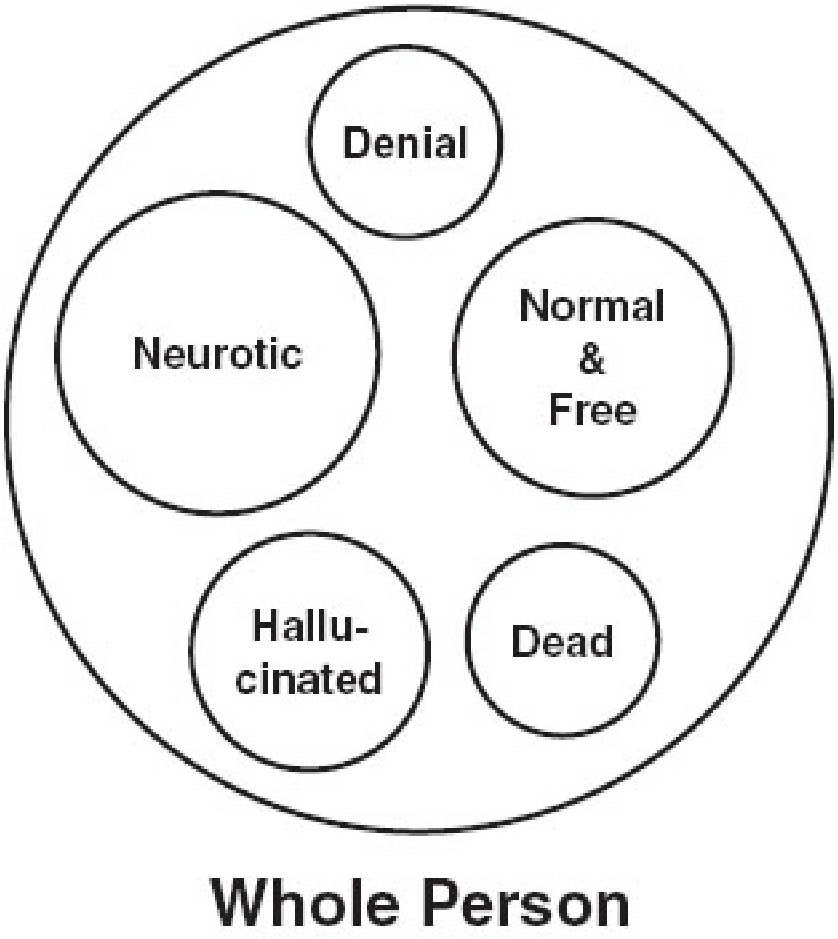

**FIGURE 3.** In practice, the patient almost always contains multiple personalities and the parts live their own lives, to a large extent. This means that different parts of the person coexist in different states. This is the analysis from holistic existential therapy of a 26-year-old female breast-cancer patient, made during the session to educate her to confront and assume responsibilities for all her "inner parts". One part of her is romanticizing and not meeting the real world (called hallucinated), another part of her is completely dead, while the largest part of her is neurotic.

## CLINICAL HOLISTIC MEDICINE

The life mission theory[4,5,6,7,8,9,15] states that everybody has a purpose of life or huge talent. Happiness comes from living this purpose and succeeding in expressing the core talent in your life. To do this, it is important to develop as a person into what is known as the natural condition, a condition where the person knows himself and uses all his efforts to achieve what is most important for him. The holistic process theory of healing[2,3,19,20] and the related quality of life theories[21,22,23] state that the return to the natural state of being is possible whenever the person gets the resources needed for the existential healing. The resources needed are "holding" in the dimensions of awareness, respect, care, acknowledgment, and acceptance with support and processing in the dimensions of feeling, understanding, and letting go of negative attitudes and beliefs. The preconditions for the holistic healing to take place are trust and the intention of the healing to take place. Existential healing is not a local healing of any tissue, but a healing of the wholeness of the person, making him much more resourceful, loving, and knowledgeable of himself, his own needs, and his wishes. In letting go of negative attitudes and beliefs, the person returns to a more responsible existential position and an improved quality of life. The philosophical change of the person healing is often a change towards preferring difficult problems and challenges, instead of avoiding difficulties in life[24,25,26,27,28,29,30,31]. The person who becomes happier and more resourceful often also becomes more healthy, more talented, and more able to function[32,33,34]. The four steps of healing the patient are (1) loving the patient, (2) winning his or her trust, (3) getting permission to give support and holding, and finally from this resourceful position, (4) processing and healing the patient[35,36,37,38,39,40,41,42,43,44].

## HEALING THE MENTALLY ILL PATIENT

The general principle in healing the mentally ill patient and inducing recovery is to process the repressed emotional pain and negative decisions. When this is done, the patients will gradually assume more responsibility for life and existence. To follow the patient step-by-step up the scale demands that the holistic physician masters the compassionate meeting of the patient in a series of specific states of consciousness (see Table 1). It is also important that the physician follows the patient as he or she moves spontaneously along the time line, and the physicians must also follow the internal shifts from one inner part ("multiple personality") to another; often the mentally ill patient will shift mood, perception, and behavior quickly and it takes a great deal of training to process the most severely ill patients. The more competent assistance the physician can give to the patient in the session, the smoother the therapy will run. Below we present a strategy for meeting the patient on the different levels of existential responsibility.

- **Step 8: No responsibility, evil, suicidal** — This patient is the most difficult to handle. It can be the anorectic patient starving herself to death, it can be the insane serial killer, or simply, the patient determined to end life by his own hand. The fundamental problem is to love this person, who has so little love for him/herself or others. Can you love a person who wants to kill you? This is the art of the physician dealing with the evil patient, the patient who has chosen to be evil[9]. Can you love a patient who destroys him- or herself? This is the problem of the many anorectic girls and psychotic incest victims often with borderline diagnoses. Can you love a person who intends to end his own meaningless life? Well, the answer to all the questions: You can if you can see and acknowledge their beautiful souls, their talents, and their gifts. So the state of unconditional love is needed to deal with the most difficult patients.
- **Step 7: Hallucinating** — This patient needs to be met in his or her own world. This takes a lot of training and will to let go of one's own ego and preconceptions of the world. But it is not impossible to visit the psychotic patient in his or her own psychotic universe and if you can do that, you can take the patient by the hand and "walk him or her out of there". This is all it takes to help an acute psychotic patient out of the psychosis. On entering into the world of the emotional pain that is caused the psychosis, it will reappear to be processed. The difficult part is to make an alliance with the patient in the psychotic state that is so strong that he or she will not let go of your hand while taking him/her through the "terrifying walls of black smoke and burning fire". That is the art of holding, when it becomes most intense.
- **Step 6: Blacking out, destroying the perception** — This step helps the patient to integrate his destruction of perception, which is what makes the blank screen to project hallucinations on. This state if often experienced by the patient as blackness and the processing is often difficult because of the lack of responsibility for perception in such events. Making the patient perceive the blackness, i.e., as "a strange shadow", and just by talking about this experience of blackness in precise words, the patient will find another perspective and soften up. The holistic medical tools of touch[36,41] are often much more efficient than anything else in this phase. Shame is often an important part of these traumas, making acceptance through touch[16] a useful tool, if the patient can accept this. Basic care is often very helpful. Sometimes advanced tools must be used[18].

- **Step 5: Denying** — Time line therapy and, even better, spontaneous regression is excellent with these patients, who now assume so much responsibility that holding and processing is often quite easy.
- **Step 4: Escaping** — This patient is almost back into reality. During the therapy, it can almost always be understood what the escape was about; most often the reason for the escape is not present anymore. Just by realizing this, much of the emotional pain is integrated.
- **Step 3: Overwhelmed** — This is the phase of emotional recovery where the patient returns to normal life and functioning. It is the recovery of character.
- **Step 2: Emotional pain** — Holistic existential standard therapy runs smoothly. It is the stage of recovery of purpose of life.
- **Step 1: Full responsibility** — The patient is back, fully alive, aware of his or her purpose of life, and able to use all his/her talents. This is the stage of optimal quality of life, health, and ability. To the frustration of some therapists, the patient will often start a new round by presenting another of the "inner parts" that needs the therapist's attention when one part (one of the "multiple personalities") is healed. This seemingly repetitive procedure, which slowly is processing many years of intense childhood suffering, often makes the therapy go on for years. Patience and deep devotion, making every moment with the patient joyful and interesting, is a must for working with the most traumatized patients.

Now, let us see how this fits in with working on the different categories of mental illnesses.

## DEPRESSION AND MANIA — AFFECTIVE DISORDERS

We become depressive when we run away from the responsibility for our own lives by describing the world, life, and ourselves as impossible. Similarly, we can escape from the world by describing ourselves as unrealistically well (the world as fantastic), which is the manic strategy. The most advanced strategy is to have both descriptions and then alternate between them at your own convenience. This is characteristic of the bipolar disorder.

Systematic and categorical denial of the value of the life inside us and in our external world leads to a life in a bleak, dark, and disconsolate world — the depressive universe. In this case, the nature of reality is the problem, including our own unbearable, but unfortunately unimprovable, personality. Thus, the problem is not that we, as responsible souls, have made some existential choices with unfortunate consequences that we now have to learn from and revise. That would be the responsible, existential perspective. Classic depression has a cognitive, an emotional, and a physical dimension. The cognitive dimension is the negative description of self, life, and reality. The emotional dimension concerns the low emotional basic mood. Depression is also associated with a number of bodily symptoms, such as reduced speed of speech and movement, waking in the early hours of the day, loss of appetite and sexual desire (libido). It is also common for depressive patients to experience chronic pain that is resistant to medication.

The depressive patient needs help to cope with being depressive and to control when the depression occurs. Instead of being "full-time depressed", controlled by the dark side, the patient can learn to become "part-time depressed", so that the patient controls when the depression should come and go, and how long it should last. By being willing to enter the depression and feel all aspects of it, and then being willing to leave it again with the knowledge from that experience, the patient can overcome his depression and no longer be enslaved by his pattern of survival. The healing process has commenced and, as the patient confronts and deals with the existential pain, he recovers.

Treatment of a depressive and psychotic patient must have due regard for the patient's safety. The therapist should handle both the structure of the patient's defenses and the underlying pain. The dark side of a psychotic or depressive patient may appear as a "helping agent", which can be very shrewd and cunning and require great skill on the part of the holistic therapist. Some patients can be very selfdestructive and the “agent” inside (the dark side) is virtually authorized to kill the patient before the existential pain is completely uncovered. This aspect demands the utmost professional expertise, which in our clinic means a referral to a psychiatrist at the slightest suspicion of specific plans for suicide, in order to ensure that the patient survives.

> **Female, aged 48 years, with bipolar affective disorder and obsessive thoughts:** Quality-of-life (QOL) conversation: Is on anticonvulsive medication, which is mood-regulating, prescribed by her psychiatrist. Manic-depressive with fixed pattern: Manic for two and a half months, depressive for two and a half months. She has been manic for nearly three months now. She describes her depressive state as follows: “My head is severed,” “I only have gloomy thoughts,” “I only think about how to get away from here with my daughter”. Major problems with ex-husband, who will not talk to her. They have had a dependency relationship, her being his obedient little dog. She still is and she feels that he is “pissing on her and that she still accepts being pissed on by him”. The authorities have granted them 12 months to become good parents; otherwise their 10-year-old son will be removed. All in all, the patient is in a miserable state, but not beyond therapeutic reach. EXERCISE 1 – in relation to ex-husband and other people: Say no, do not put up with being messed about, express your anger when you feel it, do not submit. EXERCISE 2 – win the case about your son – take responsibility for his entire parenthood and make sure that he gets what he needs. Or bring him here perhaps, so we can look at the interaction between the two of you. EXERCISE 3 – be depressed for 15 min. a day. Sit on a chair and be depressed. Find statements such as: “Everything is hopeless.” “There’s nothing good awaiting me,” and let go of them. PLAN: Rosen session once a month, with physician (SV) once a month.

This patient is in a miserable state. When you spend half your time wishing to “get away from here and take your child with you”, the situation is really serious. Since she has not killed herself and has suffered from her illness for half her life, the risk of suicide is not great. She will not be redeemed until she confronts the underlying pain that led to the negative decisions that now control her life.

> **Male, aged 55 years, with manic-depressive disorder:** QOL conversation: The patient has responded to body therapy with mania. Has a history of manic-depressive disorder going back many years. Treated by his own psychiatrist with lithium. Treated by anthroposophical doctor for 13 years with Cikorium [chicory root] and gold D 6 against mania, Terraxikum (dandelion), gold D 30 and other substances. Muscle pain throughout the body, tension headache and fatigue. On examination: Appears somewhat bleary-eyed, all trigger points tender /fibromyalgia/. Instructed to “feel, acknowledge, let go.” PLAN: Wishes to go into psychotherapy and can start here with one of our psychiatric consultants. The topic would be to take responsibility for his own feelings: Six gestalt therapy sessions, then appointment again. Then, he should be encouraged to choose the doctor he wants in future. EXERCISE: Read three good books on personal development – find a new life philosophy, ideally one that concerns responsibility.

Here the patient is merely supported in helping himself by getting a grip on the concept of responsibility in theory and in practice. We guide him, as gently as possible, into acknowledgment.

A patient who has received treatment for 13 years without any progress is likely to begin to wonder whether the treatment is working. Many alternative therapists tell their patients that the beneficial effect sets in slowly and imperceptibly, so they have to be patient. A surprisingly large number of patients undergo all sorts of peculiar courses of treatment before painfully acknowledging that they are not being helped. We believe that patients should experience being helped from the very first appointment at our clinic, including when we provide holistic treatment. If they do not, they should find another therapist. It is a general rule that if everything is exactly the same as before you had the treatment, you should not expect it to be effective in the long run either. Good therapy is effective and leads to immediate improvement, but naturally it may take a long time to resolve the problem completely. If we are initially unable to help the patient just a little bit, we doubt that we will be able to help the patient at all. Our advice to a patient asking whether acupuncture, for instance, is effective, is therefore as follows: Feel for yourself whether it is effective.

## THE CLASS OF SCHIZOPHRENIAFORM PSYCHOSIS

We can also run away from existential pain by escaping from the world in general, prompted by decisions that deny the existence of ourselves, life, and/or the world around us. This causes the classic psychosis characterized by depersonalization (I am not real), derealization (the outside world is not real), and devitalization (life is not real, but merely something mechanical and inanimate). The primary characteristic of psychosis is the lack of reality testing (i.e., poor contact with reality) and emotional blunting. Denial here goes all the way through blackness to hallucination. Occasionally, but not always, we see the split personality that led to the disease being named schizophrenia. The split implies that two different sides cannot be united in the person's character; for instance, one side is vibrant and driven by instinct and the other side is conscious, inhibited, and conscientious. According to a holistic interpretation, the split personality stems from the negative decisions.

> **Female, aged 29 years, with acute psychosis:** The patient has spontaneously entered something old and very painful. She is uncontactable, not in the present. She becomes agitated and tries to hit me (the physician) with the furniture. She then smashes up the furniture systematically in a classic catharsis /acute psychosis/. Guarded so that she does not hurt herself and kept under observation to determine appropriate treatment.

In rare cases, we come across the classic picture of "madness" where the patient is furious, evil, and very destructive. Psychosis is often a quite subtle diagnosis and fairly inconspicuous to lay people. This particularly applies to patients with borderline personality, where we sense that the patient "has a screw loose".

> **Male, aged 29 years, with borderline condition?:** QOL conversation: Very pensive as a child. Assaulted by bikers at the age of 18 years, anxiety since then. Has seen a psychologist. Degree in engineering. At the age of 20 years he was furiously angry with the system, smoked a lot of cannabis. Smashed up his room, had a psychotic episode with 5 days in closed psychiatric unit. Admitted to closed psychiatric unit again with cannabis psychosis for three weeks at the age of 21. The patient is writing his autobiography. The patient believes that he is harbouring so much pain that he is at risk of dying from it. According to his records he has suffered from depression and paranoid psychosis with delusions. He has therefore received: Zoloft [sertraline] and Zyprexa [olanzapine] for the past couple of years. Prescribe Zoloft 50 mg bid /suspected borderline/. Analysis: The patient is a truth-seeking person, who early in his life encountered the injustices of the world and raged about them. Anger and aggression are major problems here, because in my opinion the patient harbours a great deal of suppressed anger. PLAN: This patient has to restore his balance without taking a wrong turn. The patient's intelligence must be mobilised. He must stop being a destructive and maladapted element. Does a lot of reading already, but should pick relevant books that can help the patient. Perhaps books about philosophy. EXERCISE: Write down the facts of the events: what happened, how did you feel. Come back in two weeks with your autobiography, then we shall look at the way you work, so that you can obtain maximum benefit from it.

Like any other patient, the psychotic patient has certain defenses against facing and integrating the problematic gestalts. The holistic therapist has to “meet and understand” the patient and handle the patient’s defenses in an intelligent manner. Basically, they are defenses against feeling the pain, but a psychiatric patient often finds it particularly difficult to begin to work with himself, since recognition of the disorder is particularly difficult together with the stigmatization of mental illness in modern society. If modern society adopted a more relaxed attitude towards mental illness and possessed greater faith in the patients’ ability to solve their problems through personal development, it would become much easier to be mentally ill and the illness would probably last a much shorter time than is the case today, when mental illness is often for life.

The fear of mental illness is understandable as we experience that schizophrenics, for example, are a great burden on themselves and the people near them throughout their lives. Some experience has shown that 20–30% of schizophrenic persons recover spontaneously. In our view, they attain recovery when they succeed in finding a genuine and intimate relationship that provides them with sufficient support to heal the old wounds to their soul spontaneously. Treatment of depressive or psychotic patients can be difficult and protracted, but may also be simple, fast, and surprisingly painless if the patient manages to obtain a clear and precise understanding of his situation. When the patient realizes the real nature of his “opponent”, he will often experience fast, effective, and lasting recovery. Complete recovery often requires years of treatment.

## DELUSIONS

According to the “feel, acknowledge, let go” hypothesis, it is possible to help patients who can confront the pain behind their illness. Making an exact diagnosis is, therefore, not important. The important thing is to obtain an accurate understanding of the patient’s existential dilemmas. The next patient suffers from delusions. If the physician understands the problem, it is often possible to make the patient understand it as well in a short time. The patient is often hallucinative in a smart and well-hidden way.

> **Male, aged 49 years, with obsessive thoughts:** Obsessive thoughts about having to take off his shoes, before stepping on to the carpet, about killing – children and adults, etc. Wants to die. His family physician has put him on antidepressive medication, apparently with no effect. A few years ago, he ran into a 45-year-old man with his car and killed him, has felt very bad since then. We talk about the death of his father, when the patient was 12 years old, which made him think that everybody around him would die and that he, too, would soon die. Cries. EXERCISE: Write your autobiography and come to terms with your difficult relationship with death, one hour a day. Can return in a month.

The feeling of guilt is hard, difficult, and we tend to repress it. If such a feeling is repressed early in life, an event occurring later in life can create an opening that will make all the old skeletons jump out of the cupboard. The patient killed a man by accident and he already has the death of his father on his conscience (children assume responsibility for everything the adults do not), so he is now falling apart and needs to sort things out. If the patient succeeds in discovering the key problem, he will soon make progress and recover. The next patient suffers from paranoia, a very unpleasant condition with basic distrust of everything and everybody.

> **Female, aged 64 years, with paranoia:** QOL conversation: Appears somewhat paranoid, her neighbours steal her mail and do other wicked things. I think that if the patient focuses on what she is good at and the useful things she can do, then the problem with her neighbours will diminish. The patient would like to change her personality, to become more extrovert and social. We talk about the patient being able to improve. EXERCISE 1: Call more on your friends and acquaintances – the patient’s own

> suggestion. EXERCISE 2: Ignore the wicked neighbours. EXERCISE 3: Her son, whom the patient has rejected, deserves a postcard.

In this case, we chose a behavioral approach: practical exercises. The actual treatment is in exercise 3. According to our analysis, the patient becomes frightened by the wickedness that she herself is still practicing in relation to her son, who is deep down, psychologically, herself.

A classic example of mental disorder is querulous paranoia. Such a patient is running away from his or her life and is engaged in constant battle with society and the authorities; soon the patient spends all his or her time writing letters of complaint. It is a very agonizing condition and the patients are often completely unaware of their illness. In the following case report, the driving force behind the complaints is the loss of a child.

> **Female, aged 41 years, with possible querulous paranoia:** QOL conversation: Alone with her 14-year-old son. Husband left her three years ago. Three months into the last relationship she became pregnant, but gave birth to a stillborn daughter. It seems as though the patient is living in the past instead of living here and now. Will not let go of her past, which means so much to her. She is very involved in a complaint concerning compulsory treatment at a psychiatric hospital. We talk about letting go of the past /suspected querulous paranoia/. EXERCISE 1: If you want to, you can turn your back on the past and face the present and the future by asking yourself: What do I need? How can I achieve that? Do not concern yourself with the past, with writing letters to the authorities, with thinking about things that happened in the past. But you need to cry and mourn the death of your daughter, so do that. EXERCISE 2: Read “The Power of Now” by Eckhart Tolle, and other good books about living in the present. EXERCISE 3: Make a complete list of all your problems in the present, write half a page about each of them – about 20 major problems. PLAN: Next appointment in one month.

## ANXIETY

Anxiety afflicts about one in ten people in Denmark. A suitable amount of anxiety is a natural element of life, but when it grows into an uncontrollable fear of death and overshadows one’s entire existence, it becomes a psychiatric disorder.

> **Female, aged 44 years, with anxiety and grief:** First visit: Having difficulties with feelings in the form of fear of death and grief that overwhelm her. We talk about how to cope better with her strong feelings – by regarding them as an acceptable part of her. The feelings are strong, but not dangerous, and they are a part of her, which she needs. On examination: not suicidal or psychotic-depressive. She can return in two weeks if problems persist.
>
> Second visit: Abdominal pain, attacks reported to last one hour a day for a month. No fever, affective pattern apparently normal, but poorly observed. On examination: Abdomen soft with no palpable tumours, no significant tenderness except minute McBurney tenderness and diffusely around navel. Rectal exploration shows no blood, normal faeces. Pelvic examination: Vulva, vagina natural, no cervical motion tenderness of the uterus, no tenderness corresponding to adnexa. No tumours, mucous membrane smooth, sphincter tone slightly above average. Other gynaecological findings: no complaints, in particular no vaginal discharge. PLAN: To be observed to see what induces the pain – e.g. related to meals? Movement? Waking? Agitation? etc. Also faeces

> to be observed, plus urine dipsticks. Blood tests. Prescribe Voltaren [diclophenac] suppositories 100 mg as required.
>
> Third visit: She is doing much better. Barely any problems for two weeks, although anxiety is still lurking beneath the surface. She recalls that it began after a serious accident at work eight years ago. Now she can handle the anxiety when it occurs, but is still scared of dwelling on it. We shall look at that next time. Once she becomes able to enter her anxiety, she will also be able to enter other feelings, also positive ones such as love and sex, which she finds difficult at present because she feels "reserved" in relation to her boyfriend. On examination: Marked improvement. Appears happy today.

By allowing the patient to harbor her feelings and assuring her that they are in no way dangerous (on the contrary), although they may be difficult, we open the waste bin that virtually explodes, having built up an enormous pressure for years in the form of denial and repression of feelings. We perceive her abdominal pain as being psychosomatic, but since the health service is founded on biomedicine, such a diagnosis is an exclusion diagnosis, which can only be made following a thorough physical assessment. We have to rule out several somatic possibilities. But everything turns out well and the patient reaches the other side, happy and free, both of abdominal complaints and of her fear of death. As an unexpected bonus, the process lead to an improved love life.

> **Male, aged 28 years, with anxiety and headache:** Frequent anxiety, suffers from fear of death – thinks of when his heart will stop beating, does not sleep. Has started seeing a psychiatrist, who prescribed Seroxat [paroxetine] for panic disorder. Also very severe headache daily. On examination: Appears very tormented and afraid, his back is tense as a rock from os sacrum to atlas /tension headache/ /fear of death/. Cannot suppress anxiety with Alopam [oxazepam] 15 mg 3 times daily. Prescribe physiotherapy. EXERCISE: Sit down and feel the anxiety, when it emerges. Do not run away from it – otherwise it will pursue you for the rest of your life. Come back next week for conversation.

Suppressed anxiety will lead to tension, typically manifested as headache as in this patient. He is a young man, full of energy, and inclined not to repress, but to integrate, things. That is the reason why his fear of death surfaces in the first place. So a better solution would be for him to name the monster and invite it in, make friends with it, and then release it.

> **Female, aged 24 years, with anxiety:** Conversation: Acute counselling during the weekend following anxiety attack. Now composed, but worried. Watched her father stab her mother with a bread knife, when she was five years old. That incident is coming back to her now. At that time she used to think: "If only they would hold onto him, because he is sick in his head". EXERCISE: Describe every detail of the incident – everything that happened, everything you felt. When you become afraid, feel yourself as the little girl again, and look at the world from her perspective. Next appointment in two weeks, when she will bring what she has written with her. Is able to work.

When the father and mother are in a life-and-death struggle, the children suffer terrible wounds to their soul. Mercifully almost all such wounds can be healed over time.

## DISCUSSION

The concept of loss of existential responsibility and subsequent degeneration of human perception is highly complex and the eight-step responsibility-for-life scale we have presented in this paper is clearly too simple. Every mental patient contains many "inner parts" that live their own lives and need individual processing along the scale. A lot of gestalts are working in the patient's mind at the same time and all the decisions that modify perception and consciousness are there at the same time, making the patient's perception spread out all over the scale, instead of being at one single point of it. This means that a patient that can be seemingly completely normal in his/her behavior can actually be 30% hallucinative and this will only show when dealing with special persons or in specific situations. Interestingly, when sitting with a patient, you can easily feel the "insane" quality of the patient and as you work your way down the time line using the holistic medical techniques of holding and processing, inducing the spontaneous regression to the patient's traumas, you will suddenly see the patient entering a psychotic state that was there all the time. It seems fair to talk about the web of consciousness, because our mind is woven of so many perceptual gestalts, memories, decisions, and philosophical attitudes.

The question is whether a scale like the one presented is meaningful at all. We think it is, as a general scheme for the degeneration of our consciousness. In the holistic clinical practice, it serves very well as a map of states and transformations. It is very interesting that you cannot hallucinate before you have cleaned your "slate" and you do this by repressing the content of your mind so efficiently that the perception turns black. Now, on the blackness, you can add anything you want. The psychotic patient has many aspects of his/her perception repressed in the same time, but still there often is a basic orientation. You can talk to the insane person, because he or she sees you in some form, maybe severely disturbed, but you are still there and you can discuss reality. The psychotic patient will perceive reality very differently from you, but if you are into symbolic language, even the discrepancies will make fine meaning to you and by using this ability to understand, you can actually help the patient to heal.

What we are doing in these examples is not very difficult; all it takes is really the intention to be of service to the patient and to come with love or intense care, win trust, give holding, and lead the processing. Interestingly, a similar idea of levels or scales of responsibility seems to be the foundation of many systems of therapy and healing, from gestalt therapy[45] to energy healing[46] using the healer as a savior and channel of divine energy, to the new religious movements, where a scale of responsibility sometimes is used together with peculiar *Star Wars*-like axioms of reincarnation. The use of the scale of responsibility for spontaneous regression and existential healing following love, trust, and full holding (the combination of care, respect, awareness, acceptance through touch, and acknowledgment) is normally not used in gestalt therapy, healing, or religious ceremonies and seems thus to be a characteristic of holistic medicine.

In contrast to the normal belief, it is possible to help the mentally ill patients to heal if you can love them, win their trust, and get allowance to give them support and holding. Even the most psychotic schizophrenic patient can heal if you can make him or her feel the existential pain in its full depth, understand what the message of the suffering is, and let go of all the negative attitudes and beliefs connected with the disease. While the most severe schizophrenic patients, especially those with a long career in mental institutions, might take many years to heal and even be out of reach because of limited resources and lack of motivation for participation in their own healing, most mentally ill young people will benefit from existential holistic processing and many might even get healed completely. This would make it possible for them to get a good life and thus be saved from the long career as mental patients, so painful for anybody involved and close to the person, and so expensive for society.

The "trick" of helping the mentally ill patient seems to be understanding the level of responsibility the patient takes and to help him or her process the trauma and decisions that made him escape responsibility for his or her own life and destiny. A scale from free perception over emotional pain to psychic death (denial of life purpose making love impossible) further down to escape and denial to destruction of own perception and hallucination seems to be a valuable tool to understand the state of consciousness of the patient and the nature of the process of healing that the patient must go through.

## CONCLUSION

We have analyzed how responsibility, perception, and behavior decays in the pathogenesis of mental disease, the hypothesis being that mental illness is caused primarily by low existential responsibility. We have mapped the subsequent loss of responsibility into an eight-step responsibility-for-life scale: (1) full responsibility and free perception, (2) overwhelming emotional pain, (3) denial of life purpose (psychic death/ego death), (4) escaping into low responsibility perspectives, (5) denial of reality, (6) destruction of own perception, (7) freedom of hallucination, (8) suicide, unconsciousness, coma, and death. The scale seems to be a valuable tool for understanding both pathogenesis and salutogenesis, the states of consciousness a mentally sick patient must go through to recover

The scale can help the holistic physician to guide the patient through the process of assuming responsibility and recovering; in the salutogenetic process, the patient enters into an altered state of consciousness, which we call “being in holistic healing”. This happens when the patient receives unconditional love (sufficient intense care) from a physician or therapist. Being fully at the patient’s service, the physician wins the trust of the patient and gets allowance to give sufficient support and holding.

In the holistic existential therapy, old childhood traumas are re-experienced and integrated. Repressed painful emotions reappear to the surface from the patient’s consciousness and a new, more constructive, understanding of life emerges; in this process, the patient gradually lets go of negative beliefs and assumes responsibility for his or her own life.

To recover responsibility for life, the patient must rehabilitate the three fundamental dimensions of existence from “theory of talent”: (1) love including purpose of life (the life mission); (2) power of mind, feelings, and body; and (3) gender, including character and sexuality. It seems that even severely mentally ill patients can recover fully if they let go of all the negative attitudes and beliefs rising from the sufferings and, thus, causing the disease. The responsibility-for-life scale can be used on patients who need a path to assume responsibility for life, both mentally and somatically ill patients (including cancer patients).

Interestingly, just a few hours of existential holistic therapy, where the patient enters the state of holistic healing and confronts the core existential problems, seems to be of significant help, especially to the young patient with the emerging disease who has not yet been given antipsychotic drugs or been institutionalized. This gives great hope for prevention and early intervention.

## ACKNOWLEDGMENTS

This study was supported by grants from IMK Almene Fond. The quality of life research was approved by the Copenhagen Scientific Ethical Committee under number (KF)V.100.2123/91.

## References

1. Ventegodt, S., Andersen, N.J., Neikrug, S., Kandel I., and Merrick, J. (2005) Clinical holistic medicine: mental disorders in a holistic perspective. Accepted by *TheScientificWorldJOURNAL.*
2. Ventegodt, S., Andersen, N.J., and Merrick, J. (2003) Holistic medicine III: the holistic process theory of healing. *TheScientificWorldJOURNAL* **3,** 1138–1146.
3. Ventegodt, S., Andersen, N.J., and Merrick, J. (2003) Holistic medicine IV: principles of existential holistic group therapy and the holistic process of healing in a group setting. *TheScientificWorldJOURNAL* **3,** 1388–1400.
4. Ventegodt, S., Andersen, N.J., and Merrick, J. (2003) Five theories of the human existence. *TheScientificWorldJOURNAL* **3,** 1272–1276.
5. Ventegodt, S. (2003) The life mission theory: a theory for a consciousness-based medicine. *Int. J. Adolesc. Med. Health* **15(1),** 89–91.
6. Ventegodt, S., Andersen, N.J., and Merrick, J. (2003) The life mission theory II. The structure of the life purpose and the ego. *TheScientificWorldJOURNAL* **3,** 1277–1285.

7. Ventegodt, S., Andersen, N.J., and Merrick, J. (2003) The life mission theory III. Theory of talent. *TheScientificWorldJOURNAL* **3,** 1286–1293.
8. Ventegodt, S. and Merrick, J. (2003) The life mission theory IV. A theory of child development. *TheScientificWorldJOURNAL* **3,** 1294–1301.
9. Ventegodt, S., Andersen, N.J., and Merrick, J. (2003) The life mission theory V. A theory of the anti-self (the shadow) or the evil side of man. *TheScientificWorldJOURNAL* **3,** 1302–1313.
10. Jørgensen, P. (1995) Recovery and insight in schizophrenia. *Acta Psychiatr. Scand.* **92(6),** 436–440.
11. Robinson, D.G., Woerner, M.G., McMeniman, M., Mendelowitz, A., and Bilder, R.M. (2004) Symptomatic and functional recovery from a first episode of schizophrenia or schizoaffective disorder. *Am. J. Psychiatry* **161(3),** 473–479.
12. Torgalsboen, A.K. (1999) Full recovery from schizophrenia: the prognostic role of premorbid adjustment, symptoms at first admission, precipitating events and gender. *Psychiatry Res.* **88(2),** 143–152.
13. Torgalsboen, A.K. and Rund, B.R. (1998) "Full recovery" from schizophrenia in the long term: a ten-year follow-up of eight former schizophrenic patients. *Psychiatry* **61(1),** 20–34.
14. Warner, R. (1983) Recovery from schizophrenia in the third world. *Psychiatry* **46(3),** 197–212.
15. Ventegodt, S., Kroman, M., Andersen, N.J., and Merrick, J. (2004) The life mission theory VI. A theory for the human character: healing with holistic medicine through recovery of character and purpose of life. *TheScientificWorldJOURNAL* **4**, 859–880.
16. Ventegodt, S., Morad, M., Hyam, E., and Merrick, J. (2004) Clinical holistic medicine: holistic sexology and treatment of vulvodynia through existential therapy and acceptance through touch. *TheScientificWorldJOURNAL* **4**, 571–580.
17. Ventegodt, S. and Merrick, J. (2005) Clinical holistic medicine: the case story of Anna. I. Long-term effect of physical maltreatment, incest, and multiple rapes in early childhood. Submitted to *TheScientificWorldJOURNAL*.
18. Ventegodt, S., and Merrick, J. (2005) Clinical holistic medicine: advanced tools for holistic medicine — a ten-level training program for the holistic physician including controlled violence, acupressure through the vagina, and psychotropic drugs. Submitted to *TheScientificWorldJOURNAL*.
19. Ventegodt, S., Andersen, N.J., and Merrick, J. (2003) Holistic medicine: scientific challenges. *TheScientificWorldJOURNAL* **3,** 1108–1116.
20. Ventegodt, S., Andersen, N.J., and Merrick, J. (2003) The square curve paradigm for research in alternative, complementary, and holistic medicine: a cost-effective, easy, and scientifically valid design for evidence-based medicine. *TheScientificWorldJOURNAL* **3**, 1117–1127.
21. Ventegodt, S., Merrick, J., and Andersen, N.J. (2003) Quality of life theory I. The IQOL theory: an integrative theory of the global quality of life concept. *TheScientificWorldJOURNAL* **3,** 1030–1040.
22. Ventegodt, S., Merrick, J., and Andersen, N.J. (2003) Quality of life theory II. Quality of life as the realization of life potential: a biological theory of human being. *TheScientificWorldJOURNAL* **3,** 1041–1049.
23. Ventegodt, S., Merrick, J., and Andersen, N.J. (2003) Quality of life theory III. Maslow revisited. *TheScientificWorldJOURNAL* **3,** 1050–1057.
24. Ventegodt, S., Andersen, N.J., and Merrick, J. (2003) Quality of life philosophy: when life sparkles or can we make wisdom a science? *TheScientificWorldJOURNAL* **3,** 1160–1163.
25. Ventegodt, S., Andersen, N.J., and Merrick, J. (2003) Quality of life philosophy I. Quality of life, happiness, and meaning of life. *TheScientificWorldJOURNAL* **3,** 1164–1175.
26. Ventegodt, S., Andersen, N.J., Kromann, M., and Merrick, J. (2003) Quality of life philosophy II. What is a human being? *TheScientificWorldJOURNAL* **3,** 1176–1185.
27. Ventegodt, S., Merrick, J., and Andersen, N.J. (2003) Quality of life philosophy III. Towards a new biology. *TheScientificWorldJOURNAL* **3,** 1186–1198.
28. Ventegodt, S., Andersen, N.J., and Merrick, J. (2003) Quality of life philosophy IV. The brain and consciousness. *TheScientificWorldJOURNAL* **3,** 1199–1209.
29. Ventegodt, S., Andersen, N.J., and Merrick, J. (2003) Quality of life philosophy V. Seizing the meaning of life and becoming well again. *TheScientificWorldJOURNAL* **3,** 1210–1229.
30. Ventegodt, S., Andersen, N.J., and Merrick, J. (2003) Quality of life philosophy VI. The concepts. *TheScientificWorldJOURNAL* **3,** 1230–1240.
31. Merrick, J. and Ventegodt, S. (2003) What is a good death? To use death as a mirror and find the quality in life. *BMJ.* Rapid Responses, 31 October. On-line at: http://bmj.bmjjournals.com/cgi/eletters/327/7406/66#39303
32. Ventegodt, S., Merrick, J., and Andersen, N.J. (2003) Quality of life as medicine: a pilot study of patients with chronic illness and pain. *TheScientificWorldJOURNAL* **3,** 520–532.
33. Ventegodt, S., Merrick, J., and Andersen, N.J. (2003) Quality of life as medicine II. A pilot study of a five-day "quality of life and health" cure for patients with alcoholism. *TheScientificWorldJOURNAL* **3,** 842–852.
34. Ventegodt, S., Clausen, B., Langhorn, M., Kromann, M., Andersen, N.J., and Merrick, J. (2004) Quality of life as medicine III. A qualitative analysis of the effect of a five-day intervention with existential holistic group therapy: a quality of life course as a modern rite of passage. *TheScientificWorldJOURNAL* **4,** 124–133.
35. Ventegodt, S. and Merrick, J. (2004) Clinical holistic medicine: applied consciousness-based medicine. *TheScientificWorldJOURNAL* **4,** 96–99.

36. Ventegodt, S., Morad, M., and Merrick, J. (2004) Clinical holistic medicine: classic art of healing or the therapeutic touch. *TheScientificWorldJOURNAL* **4,** 134–147.
37. Ventegodt, S., Morad, M., and Merrick, J. (2004) Clinical holistic medicine: the “new medicine”, the multiparadigmatic physician and the medical record. *TheScientificWorldJOURNAL* **4**, 273–285.
38. Ventegodt, S., Morad, M., and Merrick, J. (2004) Clinical holistic medicine: holistic pelvic examination and holistic treatment of infertility. *TheScientificWorldJOURNAL* **4**, 148–158.
39. Ventegodt, S., Morad, M., Hyam, E., and Merrick, J. (2004) Clinical holistic medicine: use and limitations of the biomedical paradigm. *TheScientificWorldJOURNAL* **4**, 295–306.
40. Ventegodt, S., Morad, M., Kandel, I., and Merrick, J. (2004) Clinical holistic medicine: social problems disguised as illness. *TheScientificWorldJOURNAL* **4,** 286–294.
41. Ventegodt, S., Morad, M., Andersen, N.J., and Merrick, J. (2004) Clinical holistic medicine tools for a medical science based on consciousness. *TheScientificWorldJOURNAL* **4,** 347–361.
42. Ventegodt, S., Morad, M., Hyam, E., and Merrick, J. (2004) Clinical holistic medicine: when biomedicine is inadequate. *TheScientificWorldJOURNAL* **4**, 333–346.
43. Ventegodt, S., Flensborg-Madsen, T., Andersen, N.J., Morad, M., and Merrick, J. (2004) Clinical holistic medicine: a pilot study on HIV and quality of life and a suggested cure for HIV and AIDS. *TheScientificWorldJOURNAL* **4**, 264–272.
44. Ventegodt, S., Morad, M., and Merrick, J. (2004) Clinical holistic medicine: induction of spontaneous remission of cancer by recovery of the human character and the purpose of life (the life mission). *TheScientificWorldJOURNAL* **4,** 362–377.
45. Perls, F., Hefferline, R., and Goodman, P. (1951) *Gestalt Therapy*. Julian Press, New York.
46. Brofman, M. (2003) *Anything Can be Healed*. Findhorn Press, Findhorn, Scotland.

## BIOSKETCHES

**Søren Ventegodt, MD,** is the director of the Quality of Life Research Center in Copenhagen, Denmark. He is also responsible for a Clinical Research Clinic for Holistic Medicine in Copenhagen and is a popular speaker throughout Scandinavia. He has published numerous scientific or popular articles and a number of books on holistic medicine, quality of life, and quality of working life. His most important scientific contributions are the comprehensive SEQOL questionnaire, the very short QoL5 questionnaire, the integrated QOL theory, the holistic process theory, the life mission theory, and the ongoing Danish Quality of Life Research Survey, 1991–94 in cooperation with the University Hospital of Copenhagen and the late professor of pediatrics, Bengt Zachau-Christiansen, MD, PhD. E-mail: ventegodt@livskvalitet.org. Website: http://www.livskvalitet.org

**Niels Jørgen Andersen, MSc,** professor, Department of Innovation and Economic Organization, Norwegian School of Management. This department conducts research and provides teaching in central topics related to innovation, business development, management of global companies, business history, and economic organization. Mr. Andersen’s research activities within the Department are related to four core subjects within the discipline: business history, cooperative organizations, business development and entrepreneurship, and finally, studies of industries with a special focus on the electricity industry. He is also the dynamic chairman of the nonprofit organization, Stiftelsen Holistisk Medisin Scandinavia, which aims to support the scientific development, research, and documentation of complementary and holistic medicine in Scandinavia. E-mail: niels.j.andersen@bi.no. Website: http://www.bi.no/users/fgl93013

**Shimshon Neikrug, PhD,** is a senior lecturer in the Department of Social Work at the College of Judea and Samaria, Ariel, Israel, where he is the director of community studies. He has lectured in Social Work at Tel Aviv University and Bar-Ilan University, as well as serving as the director of the research program in the Bar-Ilan Brookdale Program. Dr. Neikrug has served as consultant to community projects in both the Palestinian Arab sector and Jewish sectors. He is the founder and chair of Yakir-Association for the Third Age. In that capacity, he has directed research projects on quality of life for both private- and public-sector organizations. His present research is on the development of informal support networks for families of children with special needs. E-mail: neikrug@bezeqint.net, Website: http://www.yosh.ac.il/socialwork/Faculty.asp?n=Neikrug_S

**Isack Kandel, MA, PhD,** is senior lecturer at the Faculty of Social Sciences, Department of Behavioral Sciences, the Academic College of Judea and Samaria, Ariel. During the period 1985–93, he served as the director of the Division for Mental Retardation, Ministry of Social Affairs, Jerusalem, Israel. E-mail: Kandeli@aquanet.co.il

**Joav Merrick, MD, DMSc,** is professor of child health and human development affiliated with the Zusman Child Development Center, Division of Pediatrics and Community Health at the Ben Gurion University, Beer-Sheva, Israel; the medical director of the Division for Mental Retardation, Ministry of Social Affairs, Jerusalem; and founder and director of the National Institute of Child Health and Human Development, Faculty of Health Sciences, Ben Gurion University of the Negev. He has numerous publications in the field of child health and human development, rehabilitation, intellectual disability, disability, health, welfare, abuse, advocacy, quality of life and prevention. Dr. Merrick received the Peter Sabroe Child Award for outstanding work on behalf of Danish Children in 1985 and the International LEGO-Prize (“The Children’s Nobel Prize”) for an extraordinary contribution towards improvement in child welfare and well being in 1987. E-mail: jmerrick@internet-zahav.net. Website: www.nichd-israel.com
